# Potential determinants of deductible uptake in health insurance: How to increase uptake in The Netherlands?

**DOI:** 10.1007/s10198-015-0745-2

**Published:** 2015-11-27

**Authors:** K. P. M. van Winssen, R. C. van Kleef, W. P. M. M. van de Ven

**Affiliations:** Institute of Health Policy and Management, Erasmus University Rotterdam, Burgemeester Oudlaan 50, 3062 PA Rotterdam, The Netherlands

**Keywords:** Voluntary deductibles, Moral hazard, Health insurance, Behavioral economics, Prospect theory, Nudge theory, D03, D11, D81, I11, I13

## Abstract

In health insurance, voluntary deductibles are offered to the insured in return for a premium rebate. Previous research has shown that 11 % of the Dutch insured opted for a voluntary deductible (VD) in health insurance in 2014, while the highest VD level was financially profitable for almost 50 % of the population in retrospect. To explain this discrepancy, this paper identifies and discusses six potential determinants of the decision to opt for a VD from the behavioral economic literature: loss aversion, risk attitude, ambiguity aversion, debt aversion, omission bias, and liquidity constraints. Based on these determinants, five potential strategies are proposed to increase the number of insured opting for a VD. Presenting the VD as the default option and providing transparent information regarding the VD are the two most promising strategies. If, as a result of these strategies, more insured would opt for a VD, moral hazard would be reduced.

## Introduction

Although fiercely debated (e.g., [24, 54]), cost sharing is an effective way to counteract moral hazard^1^ in health insurance [37, 41, 74]. One type of cost sharing is to provide the insured with the possibility to opt for a voluntary deductible in return for a premium rebate. Previous research has shown that a voluntary deductible was expected to be financially profitable for almost 50 % of the Dutch population in 2014 [65], while at the same time only 11 % of the Dutch insured opted for a voluntary deductible [67]. This discrepancy suggests that reasons other than the profitability influence the decision to opt for a voluntary deductible. The aim of this paper is twofold: (1) to identify determinants of the decision to opt for a voluntary deductible to shed light on the observed discrepancy, and (2) to provide strategies that can potentially increase the number of insured opting for a voluntary deductible. After all, if more insured would opt for a voluntary deductible, moral hazard will, ceteris paribus, be reduced.

In the next section, we elaborate on the Dutch example in which the discrepancy is observed (“The Dutch situation” section). In the “Potential determinants of the decision to opt for a voluntary deductible” section, six potential determinants of the decision to opt for a voluntary deductible are identified from the behavioral economic literature, and these determinants are discussed in order to shed light on the observed discrepancy. Subsequently, the “Potential strategies” section provides five potential strategies to increase the number of insured opting for a voluntary deductible. Finally, the implications for moral hazard are discussed in the “Implications for moral hazard” section and the conclusion is provided in the “Conclusions” section.

## The Dutch situation

The Health Insurance Act, enacted in 2006,^2^ obligates all Dutch residents to buy basic health insurance from a private insurer for community-rated premiums, which are mostly automatically deducted from the insured’s bank account [51]. By this law, a mandatory deductible requires each adult to pay the first €360 (i.e., deductible level of 2014) of healthcare expenses out-of-pocket per year. On top of the mandatory deductible, adults can opt for a voluntary deductible of one of five levels (€100, €200, €300, €400, or €500) for which they receive a premium rebate in return that is deducted from their monthly premium. Lawfully, the rebate must be equal for each insured opting for the same deductible level within the same health insurance product.^3^ In 2014, the average premium rebate for the highest deductible level was €240 and varied among insurers from €180 to €300 per individual per year. In financial terms, opting for a voluntary deductible in a specific year has been profitable for an individual if the out-of-pocket expenses under the voluntary deductible (on top of the mandatory deductible) in that year were smaller than the offered premium rebate of that year [64, 65]. Based on Dutch claims data of more than 800,000 individuals, Van Winssen et al. [65] showed that opting for the highest voluntary deductible level against the average premium rebate would retrospectively have been profitable for 48 % of the Dutch insured in 2014. Their research showed that, on average, a voluntary deductible was profitable for males up to the age of 50, for healthy insured, and for insured for whom opting for a voluntary deductible would have been profitable in previous years. They additionally show that for almost 20 % of the insured, a voluntary deductible would have been profitable in all 5 years prior to their research year, implying that for a substantial group of insured the profitability is fairly stable over the years. In real life, however, only 11 % of the Dutch insured indeed opted for a voluntary deductible in 2014 [67]. The discrepancy between the latter group and the group of insured for whom a voluntary deductible would have been profitable (e.g., 48 %) implies that determinants other than the potential financial benefit might influence the decision to opt for a voluntary deductible. Six potential determinants are identified and discussed in the next section.

## Potential determinants of the decision to opt for a voluntary deductible

### Loss aversion

A first potential explanation for the observed discrepancy between the percentage of insured (i.e., about 48 % in the Netherlands in 2014) for whom a voluntary deductible is expected to be profitable and the percentage of insured (i.e., 11 % in the Netherlands in 2014) who actually opted for a voluntary deductible, is loss aversion. Kahneman and Tversky [26] explain loss aversion by stating that ‘losses loom larger than gains’ and that ‘the aggravation that one experiences in losing a sum of money appears to be greater than the pleasure associated with gaining the same amount’. Loss aversion is denoted by *λ*, where *λ* > 1 implies loss aversion with avoidance of losses and little attention to gains and *λ* < 1 implies gain seeking with little attention to losses [70]. Tversky and Kahneman [62] estimated *λ* to be 2.25, meaning that the pain of losses is felt 2.25 times as much as the joy of gains. Attema et al. [1] on the other hand estimated *λ* in the health domain to be 1.18.

According to Kahneman and Tversky’s cumulative prospect theory (CPT) [26, 62], the overall value (*V*) of a decision (or prospect) is expressed in terms of a subjective value (*υ*), which assigns to each possible outcome (*x*) a number that reflects the subjective value of that outcome, and a decision weight (*ω*), which associates with each probability (*p*) a decision weight that reflects the impact of this probability on the overall value of the prospect. Opposed to previous studies (e.g., [12, 34]), CPT applies the principle of diminishing marginal sensitivity to both the value function and the weighting function. For decision weights, this implies an inverted S-shaped weighting function that differs for gains and losses. The outcomes are defined relative to a reference point, which implies that the value function measures the value of deviations from this reference point: either gains or losses (respectively *ω* ^+^ and *ω* ^−^)^4^ [26]. In case of a binary prospect (*p*, *x*; *y*), where the outcome is *x* with probability *p* and the outcome is *y* with probability 1 − *p* (such as is the case with opting for a voluntary deductible), the evaluation of prospects becomes [70]:

$$V\left( {p, x;y} \right) = \omega^{ + }\left( p \right) \times \left( {u\left( x \right) - u\left( y \right)} \right) + u\left( y \right)\,{\text{for pure gain prospects;}}$$

$$V\left( {p, x;y} \right) = \omega^{ - }\left( p \right) \times \left( {u\left( x \right) - u\left( y \right)} \right) + u\left( y \right)\,{\text{for pure loss prospects, and;}}$$

$$V\left( {p, x;y} \right) = \omega^{ + }\left( p \right) \times u\left( x \right) + \omega^ - \left( {1 - p} \right) \times u\left( y \right)\,{\text{for mixed prospects, where }}x { > }y.$$

Often it is assumed that the reference point in any decision is the current state of wealth, although the expected state might be the relevant reference point in some situations [72]. In case of the decision to opt for a voluntary deductible, several views and associated reference points can be considered. Table 1 shows four potential scenarios and the way they are evaluated according to CPT based upon two dimensions of the decision to opt for a voluntary deductible. The first dimension regards whether the premium for health insurance is excluded or included in the insured’s perception. The second dimension regards whether the decision is perceived as a one-stage or two-stage process. If the decision is perceived as a one-stage process, the premium rebate is integrated into the deductible amount, while if a two-stage process is perceived, the received premium rebate is separated from the deductible amount. Hershey and Schoemaker [20] and Bleichrodt et al. [5] found that one of the offered alternatives is often taken as the reference point. Schmidt [52] adds that the reference point when opting for a voluntary deductible is most likely full insurance. This would imply that not opting for a voluntary deductible seems to be the relevant reference point in each of the four scenarios in Table 1. This means that, from this reference point, the insured decides whether to opt for a voluntary deductible or to retain the reference point.

**Table 1:** Four scenarios, and the way they are evaluated according to CPT, regarding the insured’s perception of the decision (or prospect) to opt for a voluntary deductible of €500, assuming that ‘not opting for a voluntary deductible’ is the reference point

		Premium	
		Excluded	Included
Process	One-stage process	Scenario 1: Mixed prospect	Scenario 3: Loss prospect
		Probability* p* to gain €240 (*x*) Probability 1 − *p* ^a^ to lose €260 (*y*)^b,c^	Probability* p* to lose €917 (*x*) Probability 1 − *p* to lose €1417 (*y*)
		*V* (*p*, *x*; *y*) = *ω* ^+^(*p*) × *υ*(240) + *ω* ^−^(1 − *p*) × *υ*(−260)	*V* (*p*, *x*; *y*) = *ω* ^−^(*p*) × (*υ*(−1417)–*υ*(−917)) + *υ*(−917)
	Two-stage process	Scenario 2: Gain and loss prospect	Scenario 4: Loss and loss prospect
		Certainty of gaining €240 (*x*) AND Probability* p* to lose nothing (*y*) Probability 1 − *p* to lose €500 (*z*)	Certainty of losing €917 (*x*) AND Probability* p* to lose nothing (*y*) Probability 1 − *p* to lose €500 (*z*)
		*V* (1, *x*) = *υ*(240) AND *V* (*p*, *y*; *z*) = *ω* ^+^(*p*) × *υ*(0) + *ω* ^−^(1 − *p*) × *υ*(−500)	*V* (1, *x*) = *υ*(−917) AND *V* (*p*, *y*; *z*) = *ω* ^+^(*p*) × *υ*(0) + *ω* ^−^(1 − *p*) × *υ*(−500)

^a^ *p* is in all scenarios defined as the probability of staying healthy, while 1 − *p* is defined as the probability of getting sick

^b^The proposed prospects (in all scenarios) concern a simplified version (i.e., either no healthcare expenses under the voluntary deductible are incurred or healthcare expenses that exceed the voluntary deductible are incurred), while, in practice, the insured has to deal with a more continuous distribution of healthcare expenses

^c^The outcomes and premiums in all scenarios are based upon the average offered premium rebate (i.e., €240) for a voluntary deductible of €500 and the average premium (i.e., €1157) in the Dutch basic health insurance in 2014

The presence of loss aversion largely depends on the perception of the reference point. Prospects coded as losses from the reference point are affected by loss aversion. Wakker [70] emphasizes that loss aversion only concerns mixed prospects (i.e., where the outcome is either a gain or a loss) and does not affect preferences between pure gain and pure loss prospects. In that case, loss aversion is only present in scenario 1, since only this scenario regards a mixed prospect. For scenario 2, loss aversion is expected to be absent because the separate stages respectively regard a gain prospect and a loss prospect^5^ but not a mixed prospect. Scenario 3 regards a loss prospect and the two stages in scenario 4 both regard a loss prospect and therefore loss aversion is expected to be absent in these scenarios. Regarding the latter scenarios, the issue of whether the health insurance premium is perceived as an intended expenditure (i.e., in some countries, including the Netherlands, individuals are obliged to buy health insurance) and therefore not subject to loss aversion or perceived as a loss and therefore potentially subject to loss aversion, is unresolved in the scientific literature to date (e.g., [3, 4, 18, 38, 39, 58]). So from the viewpoint of scenario 1, the insured may forego the voluntary deductible since they are loss-averse and prefer the reference point (i.e., no voluntary deductible).

### Risk attitude

Risk attitude is a second potential determinant of the decision to opt for a voluntary deductible. Kahneman and Tversky [26] propose that diminishing marginal sensitivity with respect to outcomes for both gains and losses enhances risk aversion^6^ for gains and risk seeking for losses. Illustratively, individuals generally prefer a certain gain of 100 over a gain of 200 with a probability of 0.5, but if the prospects are reversed (i.e., a certain loss of 100 or a loss of 200 with a probability of 0.5), individuals prefer the latter option. However, the combination of diminishing marginal sensitivity for both the value function and the decision weighting function implies a fourfold pattern of risk attitudes: individuals are risk-averse for gains and risk-seeking for losses of moderate to high probabilities (larger than approximately 0.35) and risk-seeking for gains and risk-averse for losses of small probabilities (smaller than approximately 0.35) [62].

The insured’s objective probability of the outcomes of opting for a voluntary deductible is unknown.^7^ The probability that opting for a voluntary deductible results in a loss would be small for healthy insured. Van Winssen et al. [65] have shown that especially young males and insured for whom a voluntary deductible would have been profitable in the past have a high probability (i.e., larger than 0.65) of a positive financial result. Furthermore, their results show, based upon a combination of background characteristics of insured, that more than 40 % of the insured have a predicted probability larger than 0.65 that opting for a voluntary deductible is profitable. Note that insured might not be aware of their own probability that opting for a voluntary deductible is financially profitable. Furthermore, prospect theory shows that individuals are bad at estimating probabilities and often overestimate probabilities of rare events [72]. Additionally, determining this probability might be very complicated and may impose a high cognitive burden. Assuming scenario 1 in Table 1 and based upon the fourfold pattern of risk attitudes, it is expected that insured within the observed discrepancy (i.e., the difference between the 11 % of insured who actually opted for a voluntary deductible and the 48 % of insured for whom a voluntary deductible would have been profitable) will be risk-averse since the probability of loss is considered to be small. However, assuming scenarios 2 and 4, where the choice is considered a two-stage process, the effect of the risk attitude is unclear. Since the first stage of the decision does not involve any uncertainty, risk attitude is not expected to have any effect on the decision in that stage. In the second stage, risk aversion is expected such as in scenario 1 because the risk of a loss remains small. However, the combined effect of both stages is unknown. Assuming scenario 3, where the decision always results in a loss, risk-seeking behavior is expected. Gorter and Schilp [16] support the notion that risk aversion potentially plays a role in the decision to opt for a voluntary deductible by showing that risk preferences (e.g., financial risk tolerance, smoking and drinking behavior) have a significant positive effect on the choice for a voluntary deductible. Rice [46] emphasizes that the degree of risk aversion alone cannot explain individuals’ preference for low deductibles and that loss aversion remains an important determinant. Additionally, several studies have shown that presenting individuals with prospects within an insurance context may enhance risk aversion. Schoemaker and Kunreuther [53] report that, although mathematically equivalent, 45 % of the respondents preferred a zero deductible option presented in an insurance context, while only 13 % preferred this option outside the insurance context. Hershey et al. [19] demonstrate a similar result and state that individuals are more risk averse under the insurance formulation than under the gamble formulation of the same prospect. Since the decision to opt for a voluntary deductible is considered within an insurance context, these studies indicate that risk aversion may be more pronounced than mentioned before. In sum, the effect of risk attitude on the decision to opt for a voluntary deductible is largely unclear and depends strongly on the scenario. For scenario 1, risk aversion is expected, which might explain why the insured forego deductibles, while for scenario 3, risk-seeking behavior is expected, which would predict that insured do opt for a deductible.

### Ambiguity aversion

Ambiguity aversion^8^ is a third potential determinant of the decision to opt for a voluntary deductible and has been incorporated into CPT. According to Ellsberg [10] ambiguity regards ‘the nature of one’s information concerning the relative likelihood of events’, which depends on ‘the amount, type, reliability and ‘unanimity’ of information’. This gives rise to ‘one’s degree of confidence in an estimate of relative likelihoods’. Frisch and Baron [13] add that this uncertainty about probabilities is created by missing information that is relevant and could be known. Ambiguity aversion captures individuals’ preferences for prospects with known probabilities over prospects with unknown probabilities and was first presented by Ellsberg [10]. In a hypothetical experiment, individuals were confronted with two urns. The first urn contained 100 red and black balls in an unknown ratio and the second urn contained exactly 50 red and 50 black balls. The majority of respondents preferred to bet on either red or black in urn two rather than in urn one, indicating ambiguity aversion. Ritov and Baron [47] show the presence of ambiguity aversion in healthcare in a study on children’s vaccination, where the vaccination reduces the risk of dying from a specific disease, but simultaneously might have adverse health effects. When ambiguity about the risk of adverse health effects was caused by missing information (i.e., a child had a high risk or no risk of adverse effects, but it was impossible to find out which) individuals were more reluctant to vaccinate, indicating ambiguity aversion. In most experiments on ambiguity aversion, respondents had to choose between two situations: one with known probabilities and another with unknown probabilities. In case of opting for a voluntary deductible, a comparison with known probabilities is absent. Chow and Sarin [9] conducted several experiments concerning ambiguity aversion under comparative and non-comparative conditions and conclude that the ambiguity effect exists under both conditions, but that it is significantly reduced in the non-comparative condition. This indicates that ambiguity aversion may actually influence the decision to opt for a voluntary deductible, even though a comparison with known probabilities lacks. Ellsberg [10] adds that individuals often perceive the status-quo as the situation with low variation and that ambiguities of the new situation are more salient than those of the current situation. Therefore, when deciding to opt for a voluntary deductible, ambiguity aversion might create a preference for the current situation. This causes insured without a voluntary deductible not to opt for a voluntary deductible in the next year even if this would result in the same (or a better) expected value. Note that from ambiguity aversion it follows that individuals will value provision of any information that reduces their ambiguity, even if it will not change their decision, while standard economic theory predicts that information is only demanded if it affects the decision [7].

Several studies argue that a (psychological) driver of ambiguity aversion is found in the competence hypothesis that states that individuals prefer to bet on their beliefs in situations where they feel knowledgeable, and prefer to bet on chance when they feel ignorant [17, 29, 63]. Several researchers show that insured have limited knowledge about their health insurance [22, 44] and others add that individuals misunderstand complex price schedules including premiums and cost-sharing arrangements [2, 32, 35]. Additionally, estimating the probability that a voluntary deductible would be financially profitable might be complex and might impose a high cognitive burden. This could especially be the case for individuals with low levels of numeracy and/or health literacy. Based upon these studies, individuals’ limited knowledge about health insurance could indicate that (in)competence affects the degree of ambiguity aversion for the decision to opt for a voluntary deductible. In sum, since probabilities regarding the profitability of voluntary deductible are absent, ambiguity aversion (partially through incompetence) might explain why insured forego deductibles.

### Debt aversion

A fourth potential determinant of the decision to opt for a voluntary deductible is debt aversion, which stems from mental accounting theory [58, 59]. Thaler defines mental accounting as ‘the set of cognitive operations used by individuals and households to organize, evaluate, and keep track of financial activities’ [59]. The theory incorporates CPT and provides a better understanding of the psychological processes that underlie choices and decisions. Prelec and Loewenstein [43] build upon Thaler’s theory and predict strong debt aversion because individuals establish mental accounts that create linkages between consumption and payments. Debt aversion in their work is defined by individuals’ preferences to prepay for consumption and to get paid for work after completion. Individuals dislike the feeling of ‘having the meter running’ and prefer flat-rate pricing schemes even if they pay more for the same usage [43, 59]. The latter is called the flat rate bias and can be illustrated by a preference for unlimited Internet access at a fixed monthly price over paying per megabyte. Prelec and Loewenstein [43] provide two motives why individuals are inclined to prepay for a product. Firstly, individuals hope to enjoy the product untroubled from payment concerns and secondly, individuals want to avoid the unpleasant experience of paying for consumption that has already been enjoyed.

Debt aversion firstly predicts that insured dislike paying for healthcare after consumption and secondly that insured prefer flat-rate pricing schemes (e.g., health insurance) to payment decoupling. This makes debt aversion relevant for the decision to opt for a voluntary deductible. Debt aversion could prevent insured from opting for a voluntary deductible since, if the insured opts for a voluntary deductible, healthcare is paid for after consumption, while if the insured does not opt for a voluntary deductible, a flat rate is paid in advance. Overall, due to the debt that results from consuming healthcare when having a voluntary deductible, insured might forego deductibles.

### Omission bias

Omission bias is a fifth potential determinant of the decision to opt for a voluntary deductible. Samuelson and Zeckhauser [50] introduced the status quo bias that describes individuals’ tendency of ‘doing nothing or maintaining one’s current or previous decision’. Ritov and Baron [48], however, state that two claims are embedded in this bias: firstly, individuals prefer to keep the current state and secondly, individuals are reluctant to take action to change this state. The latter is called omission bias. Ritov and Baron [48] explain status quo bias by the fact that changing the status quo requires an act, while keeping the status quo requires only an omission. Through three experiments they show that the omission bias was present in choice whether the status quo was changed by action or not. Furthermore, they demonstrate that no consistent status quo bias was found in choice when both choices did (not) involve an action. This result corresponds to norm-theory where Kahneman and Miller [27] state that omissions are considered the norm, while commissions are compared to what would have happened if nothing had been done. So, regardless of the outcome, omissions are evaluated as neutral, where commissions are evaluated as negative if the outcomes are worse and evaluated as positive if the outcomes are better than the expected outcome of inaction.

A potentially underlying factor of omission bias is decision fatigue, which means that individuals tire from making decisions in general [72]. A second potentially related factor to omission bias concerns transaction costs [55]. Transaction costs regard the time and effort that it takes to choose a plan with or without a voluntary deductible. Another potentially related factor is regret avoidance, which implies that whenever choice can induce regret, individuals have an incentive to eliminate choice [57]. Regret avoidance helps explain individuals’ preference for first dollar coverage, since many individuals find decisions that involve trade-offs between healthcare and money unpleasant [57]. Thaler [57] considered the following example: for their child, a couple has to decide on taking a diagnostic test that costs *x*. A small risk exists that the child has a serious disease that can only be treated if detected early. The couple will certainly experience regret if they decide not to test the child and he/she is found to have the disease. If the test is performed and shows the likely negative result, the couple may regret the expenditure, especially if it was expensive relative to their wealth. These psychic costs could be avoided if all healthcare consumption is prepaid and no decision (i.e., act) is required.

Decision fatigue may affect the decision to opt for a voluntary deductible, since insured might just be tired from making all kinds of (financial) decisions and therefore decide to renew their current plan (i.e., the plan with(out) a voluntary deductible). Furthermore, transaction costs may affect the decision to opt for a voluntary deductible since insured would want to avoid these costs and therefore renew their current plan (i.e., the plan with(out) a voluntary deductible). Regret avoidance may affect the decision to opt for a voluntary deductible since insured might not want to take the risk of having to regret the decision (not) to opt for a voluntary deductible if healthcare expenses that exceed (or stay below) the deductible amount are incurred. Additional to these direct effects, these factors might also indirectly effect the decision to opt for a voluntary deductible. After all, if an insured has opted for a voluntary deductible, he needs to make more and more complex decisions regarding the usage of healthcare services (e.g., when and where to seek care and how much these services cost) while little support for making these decisions is available. With this in prospect when opting for a voluntary deductible, omission bias might also indirectly prevent insured from opting for a voluntary deductible. In short, omission bias (and related to that, decision fatigue, transaction costs and regret avoidance) may directly and indirectly affect the decision to opt for a voluntary deductible since for most insured (i.e., those insured without a voluntary deductible) it requires an act to change their current plan to a plan with a voluntary deductible, which they are reluctant to do.

### Liquidity constraints

A sixth potential determinant of the decision to opt for a voluntary deductible is the fear of encountering liquidity problems. Gorter and Schilp [16] hypothesize that consumption commitments (e.g., mortgage payments) explain the low percentage of Dutch insured opting for a voluntary deductible. Additionally, several studies researched the impact of liquidity constraints on risk attitude and loss aversion. Firstly, Chetty and Szeidl [8] conclude that consumption commitments, since they are costly to adjust (e.g., mortgage payments can only be adjusted by moving), increase risk aversion for small and moderate stakes. For example, if an individual is forced to reduce his expenditure by 10 % and has precommitted 50 % of his income, he must reduce spending on discretionary items by 20 %. Since the precommitted expenditure is not freely adjustable, the utility curvature is greater than if it would be adjustable as to amplify risk aversion. Secondly, Novemsky and Kahneman [38] state that for consumers who maintain a tight budget, the purchase of a good that was not budgeted for is associated with giving up some other good (i.e., either consumption or savings). This is then evaluated as a loss, which is consistent with the finding of Wicker et al. [71] that there is more loss aversion when a greater proportion of money is designated for necessities. These studies indicate that liquidity constraints could be closely related to other determinants that are identified within this paper, such as risk attitude and loss aversion. Thirdly, Sydnor [56] investigated if liquidity constraints explain the preference for low deductibles in home insurance. Though this was not the case, it could be interesting to study whether this holds for the health insurance market.

Due to liquidity constraints, insured might not opt for a voluntary deductible because they may be unable or may fear to be unable to pay the deductible amount if healthcare is consumed.^9^ Furthermore, liquidity constraints are expected to increase risk aversion and loss aversion and thereby (negatively) affect the decision to opt for a voluntary deducible.

### Overview of potential determinants

This section discusses in short the effect of the six potential determinants on the decision to opt for a voluntary deductible. Note that the different determinants are not per definition independent, and could be closely related (e.g., liquidity constraints could be related to risk aversion). Loss aversion is only expected to make insured forego voluntary deductibles in scenario 1 from Table 1. For scenario 1, the fourfold pattern of risk attitudes furthermore predicts risk-averse behavior, while the effect for scenarios 2 and 4 (i.e., the two-stage scenarios) is unclear since in the first stage uncertainty plays no role while risk aversion is expected for the second stage. Regarding scenario 3, risk-seeking behavior is expected that may encourage insured to opt for a voluntary deductible. Furthermore, irrespective of the scenario, ambiguity aversion may arise since the probability distribution of healthcare expenses is largely unknown, which may explain why insured forego deductibles. Since in case of a voluntary deductible healthcare is consumed first and paid after, debt aversion may explain why insured do not opt for a voluntary deductible. Omission bias is seen as a fifth potential determinant since individuals are reluctant to take action to change their current plan, which is necessary for the uptake of deductibles. Finally, liquidity constraints are expected to influence the decision to opt for a voluntary deductible and increase both risk aversion and loss aversion. In general, it is expected that especially in scenario 1 of Table 1, insured would not be inclined to opt for a voluntary deductible since the six potential determinants all negatively affect the overall value of opting for a voluntary deductible compared to not opting for a voluntary deductible. In scenarios 2, 3, and 4, some of the determinants are not or less relevant, i.e., loss aversion is expected to be absent in those scenarios and the effect of risk attitude is unclear in scenarios 2 and 4. Therefore, the overall effect of these scenarios on the decision to opt for a voluntary deductible is unclear.

Which of the proposed scenarios is adopted by insured in practice is unknown. For two reasons, however, we believe that scenarios 1 and 2 are most likely to be adopted. The first reason is that we suspect that (Dutch) insured do not include the premium in their decision. For example, taking out health insurance is mandatory in the Netherlands and the decision to opt for a voluntary deductible is a subsequent decision that may not be directly related to the fact that a premium has to be paid for health insurance itself. The second reason is that the premium is mostly paid on a monthly basis, while the voluntary deductible concerns a yearly amount. This might make integrating the health insurance premium into the decision to opt for a voluntary deductible difficult for insured.

## Potential strategies

Based upon the six potential determinants of the decision to opt for a voluntary deductible, this section discusses five potential strategies that could increase the number of insured opting for a voluntary deductible. While discussing these strategies, we will incorporate nudge theory as proposed by Thaler and Sunstein [61]. The idea behind nudging is to move individuals in directions that will make their lives better without forcing them. They consider a nudge to be ‘any aspect of the choice architecture that alters people’s behavior in a predictable way without forbidding any options or significantly changing their economic incentives’ [61]. Nudges are not considered to be mandates and should be easy and cheap to avoid. An illustrative example of a nudge is putting fruit at eye level in a school canteen to make children eat healthier, while entirely banning junk food would not be considered a nudge. Note that not all proposed strategies can be considered a nudge and that within this paper only strategy one and three are considered a nudge. This will be further discussed in the subsequent sections. Table 2 shows how the five different strategies affect the determinants of the decision to opt for a voluntary deductible.

**Table 2:** Summary table of the determinants regarding the decision to opt for a voluntary deductible (VD) and the way they are affected by the different strategies

	Strategy 1	Strategy 2	Strategy 3	Strategy 4	Strategy 5
	Default option	Information regarding the voluntary deductible	Information regarding healthcare expenses	No-claim rebate	Saving for healthcare
Loss aversion	If the reference point is ‘opting for a VD’, loss aversion is eliminated. If ‘not opting for a VD’ is the reference point, then loss aversion remains the same	–	–	Loss aversion could be reduced depending on whether loss aversion occurs for the premium	–
Risk attitude	If the reference point is ‘opting for a VD’: risk seeking. If ‘not opting for a VD’ is the reference point: risk aversion	–	–	–	–
Ambiguity aversion	–	Increased competence and decreased ambiguity aversion	Reduced effect of ambiguity aversion	–	–
Debt aversion	–	–	–	Reduced effect of debt aversion	Reduced effect of debt aversion
Omission bias	Omission bias remains, but causes the insured to retain the VD	–	–	–	–
Liquidity constraints	–	–	–	–	Reduced effect of liquidity constraints

### Default option

A first promising strategy for increasing the number of insured opting for a voluntary deductible is to present the voluntary deductible as the default option. This implies that when buying insurance, the plan includes by default a voluntary deductible for the associated premium. The plan would not be mandatory because insured can commute the voluntary deductible for an increase in premium. Table 3 shows the insured’s perception of the voluntary deductible as the default option. According to the literature, this strategy is expected to increase the number of insured opting for a voluntary deductible [25, 31, 36, 60, 61] since it potentially affects three determinants from the theoretical framework. Note that this strategy intends to shift the reference point from ‘not opting for a voluntary deductible’ to ‘opting for a voluntary deductible’. Firstly, the effect of loss aversion would diminish since, assuming that ‘opting for a voluntary deductible’ is the reference point, ‘not opting for a voluntary deductible’ is a pure loss prospect (i.e., the deductible has to be commuted for an increase in premium). In case of a pure loss prospect, loss aversion is absent [70] and therefore has no effect on the decision to opt for a voluntary deductible if a voluntary deductible is the default option. Secondly, with ‘opting for a voluntary deductible’ as the reference point, risk seeking behavior is expected since commuting the voluntary deductible implies a certain loss. Thirdly, with this strategy, the insured is inclined to retain the voluntary deductible due to omission bias (and decision fatigue and transaction costs). If a default option is set, it is expected that more insured would opt for a voluntary deductible than under an opt-in design. Therefore, making the voluntary deductible the default option can be considered a strong nudge. Furthermore, the nudging power of the default option will be reinforced if the option comes with some implicit or explicit suggestion that it represents the norm, which is related to norm-theory, or the recommended course of action [61]. An example of the effect of default options can be found in MediShield (a basic catastrophic illness insurance scheme) in Singapore. In 1990, with the introduction of MediShield, the Singapore government wanted to ensure that as many individuals as possible would be covered by this plan. In order to reach this goal, they implemented an opt-out scheme where everyone would be automatically enrolled. As a result of this, the overall coverage for MediShield raised from 51 % in 1990 to 88 % in 2012 [33].

**Table 3:** The insured’s perception and the associated value function regarding the voluntary deductible for strategy one (i.e., the default option) and strategy four (i.e., a no-claim rebate). Presented are the perceptions for scenario 1 from Table 1, but they could be applied to the other scenarios as well

Opting for a voluntary deductible	Not opting for a voluntary deductible
Strategy 1—Present the voluntary deductible as the default option	
Probability *p* to lose nothing (*x*) Probability 1 − *p* to lose €500 (*y*)	Certainty of a premium increase of €240 (*x*)
*V* (*p*, *x*; *y*) = *ω* ^+^(*p*) × *υ*(0) + *ω* ^−^(1 − *p*) × *υ*(−500)	*V* (1, *x*) = *υ*(−240)
Strategy 4—Offer a voluntary deductible in the form of a no-claim rebate	
Probability *p* to gain €240 (*x*) Probability 1 − *p* to pay €260 (*y*) too much	Certainty to lose nothing (*x*)
*V* (*p*, *x*; *y*) = *ω* ^+^(*p*) × *υ*(240) + *ω* ^−^(1 − *p*) × *υ*(−260)	*V* (1, *x*) = *υ*(0)

### Provision of information regarding the voluntary deductible

A second promising strategy to increase the number of insured opting for a voluntary deductible is to provide insured with information regarding the voluntary deductible. According to the literature, this strategy is expected to increase individuals' competence [35]. Through the increase in competence, the effect of ambiguity aversion on the decision to opt for a voluntary deductible could be reduced [17, 29, 63], which could result in a higher uptake of voluntary deductibles. The information could for instance concern the functioning of the voluntary deductible.^10^ The information could elucidate that a voluntary deductible results in both a premium rebate and a risk that out-of-pocket payments have to be made and that the profit is the balance of these two. The information could furthermore describe the relation between the voluntary deductible and other cost-sharing arrangements such that individuals can better estimate their expected out-of-pocket expenses due to the voluntary deductible (and thus whether opting for a voluntary deductible will be profitable). For example, Van Winssen et al. [65] show that over 40 % of the Dutch insured had healthcare expenses even below the mandatory deductible of €360. For those insured, opting for a voluntary deductible would be profitable, but they need to know how the voluntary deductible and other cost-sharing arrangements relate in order to consider opting for a voluntary deductible. Finally, Reitsma-van Rooijen et al. [45] show that Dutch insured avoid the GP because of the mandatory deductible, while GP costs are exempted from the deductible. Apparently, these individuals are not aware of the fact that these healthcare services are exempted from the deductible. Therefore, information could address the exempted healthcare services. Note that all information should be understandable because if individuals are provided with information only an expert would know how to use, incompetence actually increases [15].

### Provision of information regarding healthcare expenses

A third potential strategy to increase the number of insured opting for a voluntary deductible is to provide insured with information regarding their healthcare expenses.^11^ For instance, the information could show the number of previous years that opting for a voluntary deductible would have been profitable. Van Winssen et al. [65] show that the more (recent) years the voluntary deductible would have been profitable in the past, the larger the probability that opting for a voluntary deductible would be profitable in the upcoming year. Furthermore, insured could be provided with an objective predicted probability that opting for a voluntary deductible would be profitable based upon background characteristics such as age, gender, and chronic illness, such as Van Winssen et al. [65] have estimated. Finally, insurers could provide insured with an up-to-date overview regarding their past healthcare expenses.

Based upon the theoretical framework in the “Potential determinants of the decision to opt for a voluntary deductible” section, this strategy is expected to directly influence the effect of ambiguity aversion on the decision to opt for a voluntary deductible since information on the outcome probability of the voluntary deductible is provided. Note that Ellsberg [10] and Fox and Weber [15] state that the amount, type, reliability, and unanimity of the information should be considered when providing the information to insured to best reduce the effect of ambiguity aversion on the decision to opt for a voluntary deductible. Wakker et al. [69] studied the effect of statistical information on the choice of insurance that covers a deductible and show that the value of the options that give rise to ambiguity aversion decreased rather than increased when ambiguity reduced. They state that probably the more familiar option is preferred over the option with known probabilities, which could be in accordance with Ellsberg’s [10] notion that ambiguity aversion favors the status quo. Additionally, their results showed that the provision of statistical information enhanced adverse selection, which for health insurance might be undesirable from the societal perspective [49]. In a study by Kling et al. [30], a random sample of participants were sent a personal letter that explained the costs of their current drug plan, the cheapest comparable plan, and the savings they could realize by switching plans. Another random sample received generic brochures regarding the different plans. The results show that the personal letters appear to have nudged more individuals to pick lower-cost plans and the overall switching rate was 10 percentage- points higher than among the participants who received the brochures [61]. These results could give an indication that providing insured with information regarding their healthcare expenses and the savings they could realize by opting for a voluntary deductible could potentially increase uptake of voluntary deductibles.

### No-claim rebate

A fourth potential strategy to increase the number of insured opting for a voluntary deductible is to present the voluntary deductible in the form of a ‘no-claim rebate’.^12^ In case of a no-claim rebate, the insured pays a premium for health insurance and receives a fixed amount of money (i.e., the no-claim rebate) at the end of the year if no healthcare expenses are incurred. If healthcare expenses are incurred, the insured receives no rebate. In other words, compared to a situation with a voluntary deductible, insured pay the full premium (i.e., they do not receive a premium rebate that they would have received if they had opted for a voluntary deductible) and receive a no-claim rebate equal to the amount of the original voluntary deductible (i.e., €500) if no healthcare expenses are incurred. Assuming the Dutch voluntary deductible of €500, the premium increase would equal €260 and the potential rebate would be €500.^13^ Compared to the current design of the voluntary deductible, the potential loss (i.e., €-260, which is equal to the premium increase) and gain (i.e., €240, which is equal to the no-claim rebate minus the premium increase) are essentially unchanged, but integrated explicitly. Table 3 shows the insured’s perception of the voluntary deductible in the form of a no-claim rebate. According to the literature, this measure is expected to increase the number of insured opting for a voluntary deductible [25] since it potentially affects two determinants from the theoretical framework. Firstly, the increase in loss aversion due to the increase in premium is expected to be small because of diminishing marginal sensitivity. The effect on loss aversion depends however on whether loss aversion for the premium is experienced. Secondly, this strategy could reduce the effect of debt aversion on the decision to opt for a voluntary deductible since the insured is not in debt with the insurer, but the insurer is potentially in debt with the individual. Furthermore, this strategy could reduce the effect of debt aversion on the decision to opt for a voluntary deductible, since the insured pays for healthcare expenses ex-ante instead of ex-post.

### Saving for healthcare

A fifth potential strategy to increase the number of insured opting for a voluntary deductible is to offer a savings account in combination with a voluntary deductible. Health Savings Accounts (HSAs) are increasingly popular in the USA, Singapore, South Africa, and China, but have different aims and designs [23]. In the USA, HSAs are combined with high deductible health plans, which is similar to the strategy proposed here. Note, however, that the deductible amounts in the USA are larger (i.e., a plan must have a minimum deductible of €1074^14^ for individuals and €2148 for families in 2015 to be HSA-eligible with a maximum limit on out-of-pocket spending of €5329 for individuals and €10,658 for families) than the amounts in the Netherlands (i.e., the voluntary deductible amounts range from €100 to €500). Similar to the HSAs, we propose to deposit the premium rebate upon a savings account allowing the insured to use the (earmarked) account balance for the voluntary deductible. Literature on savings behavior shows that individuals have self-control problems [28], meaning that individuals have difficulty with not spending their money on other purposes [40]. An earmarked savings account could mitigate this lack of self-control by serving as a precommitment strategy [57]. Table 4 provides a potential way to finance the savings account in case the insured opted for a voluntary deductible of €500 and deposited the premium rebate of €240 on the savings account (column 2). A maximum of €1200 is saved during, for example, 5 years (column 3). Out-of-pocket payments due to the voluntary deductible are paid from the savings account (e.g., €25 in the first year, column 4). Column 5 shows the account balance at the end of each year (i.e., €215 in the first year). In the coming years, the financing of the account continues in the same way.

**Table 4:** Example of financing the savings account in case of a voluntary deductible of €500 with an associated premium rebate of €240 (in euros)

	Premium rebate	Total premium rebate on savings account	Healthcare expenses under the voluntary deductible	Account balance at the end of the calendar year
Year 1	240	240	25	215
Year 2	240	480	120	335
Year 3	240	720	500	75
Year 4	240	960	175	140
Year 5	240	1200	0	380

According to the theoretical framework in the “Potential determinants of the decision to opt for a voluntary deductible” section, there are two reasons to expect that this strategy will increase the number of insured opting for a voluntary deductible. Firstly, the savings account could serve as a prepayment vehicle that diminishes the attenuation of the payment on the pleasure of consumption, which could reduce the effect of debt aversion. Secondly, the earmarked savings account serves as a consumption commitment especially for out-of-pocket payments due to the voluntary deductible, which could reduce the effect of liquidity constraints. In the USA, savings into the HSA are encouraged by tax advantages. This could also be considered for the savings account as described here to encourage insured to save for potential out-of-pocket payments due to the voluntary deductible.

## Implications for moral hazard

The previous section discussed five potential strategies to increase the number of insured opting for a voluntary deductible and consequently to reduce moral hazard. Behavioral economics helps to explain the demand for voluntary deductibles, but also tells us that the design of the voluntary deductible could influence healthcare usage [43]. To indicate the net effect on moral hazard a crucial question is: in what way does the design of the voluntary deductible (within the different strategies) influence the moral hazard reduction (assuming that the strategies increase the number of insured opting for a voluntary deductible)? Note that the reduction of moral hazard as a result of the voluntary deductible could regard both low-value and high-value care. The RAND Health Insurance Experiment showed, however, that on average the moral hazard reduction had no significant effect on most of the studied health indicators [6].

By presenting the voluntary deductible as the default option and by providing insured information regarding the voluntary deductible or regarding their healthcare expenses (i.e., strategies 1, 2, and 3), the voluntary deductible in itself is unchanged and therefore the individual moral hazard reduction due to the voluntary deductible is unchanged. However, since, as a result of these strategies, an increase in the number of insured opting for a voluntary deductible is expected [25, 31, 35, 60, 61], an increase in the total moral hazard reduction is expected as well. When presenting the voluntary deductible as a no-claim rebate (i.e., strategy 4), the effect on the individual moral hazard reduction is unclear, since in case of a no-claim rebate insured only have the foresight of a potential rebate and do not experience actual out-of-pocket payments as they do with the current design of the voluntary deductible. It is unknown how many more insured would have to opt for a voluntary deductible in the form of a no-claim rebate in order to let the total moral hazard reduction increase. To determine the effect of offering a savings account in combination with a voluntary deductible (i.e., strategy 5) on moral hazard, it would be necessary to know whether the moral hazard reduction differs between actual out-of-pocket payments (i.e., the current design) and expenses from a savings account (i.e., strategy 5). Since the money is earmarked for healthcare expenses, individuals might be more eager to spend saved money than out-of-pocket money. Therefore, what happens to the account balance at the end of the year is essential (e.g., transmitted to next year, paid to insured or lapsed). A related (and yet unanswered) question to this is how individuals value the account balance at the end of the year, taking into account discounting of money over time [40]. As with strategy 4, it is unknown how many more insured would have to opt for a voluntary deductible as a result of combining it with a savings account to let the total moral hazard reduction increase. Overall, each strategy is intended to increase the number of insured opting for a voluntary deductible, which, ceteris paribus, increases the total moral hazard reduction due to the voluntary deductible. However, the effect of (some of) the strategies on the individual moral hazard reduction and consequently on the total moral hazard reduction needs further research.

## Conclusions

Previous research shows that a large discrepancy exits between the percentage of insured for whom a voluntary deductible is expected to be profitable (i.e., about 48 % in the Netherlands in 2014) and the percentage of insured who actually opt for a voluntary deductible (i.e., 11 % in the Netherlands in 2014). If more insured would opt for a voluntary deductible, a larger reduction of moral hazard could, ceteris paribus, be reached. In this paper, six determinants of the decision to opt for a voluntary deductible are identified: loss aversion, risk attitude, ambiguity aversion, debt aversion, omission bias, and liquidity constraints. Subsequently, five potential strategies to increase the number of insured opting for a voluntary deductible are proposed: (1) present the voluntary deductible as the default option, (2) provide insured with information regarding the voluntary deductible, (3) provide insured with information regarding their healthcare expenses, (4) present the voluntary deductible in the form of a no-claim rebate, and (5) combine the voluntary deductible with a savings account. We believe that implementing the voluntary deductible as the default option and providing insured with information regarding the functioning of the voluntary deductible are the two most promising strategies to increase uptake of voluntary deductibles and to reduce moral hazard. Regarding the other strategies, further research on their effect on the moral hazard reduction would be necessary before implementing such strategies.

## Footnotes

1 Moral hazard refers to the change in health behavior and consumption caused by the fact that the health insurer reimburses the costs [14, 73].

2 Since the possibility to opt for a voluntary deductible has already existed for a decade, we do not expect that unfamiliarity with the deductible would be a large contributor to the explanation of why the insured forego deductibles. However, the percentage of insured with a voluntary deductible has increased considerably in these years (i.e., from 5 % in 2006 [66] to 11 % in 2014 [67]).

3 Additionally, insurers may offer the insured who have opted for a voluntary deductible in previous years a different premium rebate. However, in practice, this does not happen.

4 The overall value of a prospect in CPT differs from that in expected utility theory (EUT) where choices under uncertainty are evaluated by their expected utility [68]. The main deviations from CPT with respect to EUT are the dependence upon a reference point, probability weighting and loss aversion [1]. We prefer to use CPT since it proves to have more descriptive validity than EUT.

5 We define a mixed prospect as a prospect that involves a gain and a loss and do not regard zero to be a gain. Therefore, we do not regard the second stage of scenarios 2 and 4 as mixed prospects but respectively as a gain and loss prospect.

6 For the definition of risk aversion see [42].

7 Obviously, chronically ill insured are aware of their probability since they know for certain that opting for a voluntary deductible is not profitable for them under the current design. However, the aim of this paper is to shed light on the discrepancy between the low percentage of insured who do opt for a voluntary deductible and the high percentage of insured for whom opting for a voluntary deductible is expected to be profitable. Opting for a voluntary deductible is not expected to be profitable for chronically ill and therefore we do not aim to provide determinants of their decision to opt for a voluntary deductible.

8 Ambiguity aversion is sometimes also referred to as uncertainty aversion.

9 Liquidity constraints may also encourage insured to opt for a voluntary deductible since the premium rebate reduces the monthly premium and relieves liquidity constraints.

10 It is assumed that individuals are aware of the fact that they can opt for a voluntary deductible at all.

11 Note that this strategy could also provide insured with information regarding how unattractive it would be for them to opt for a voluntary deductible, making them less inclined to opt for a voluntary deductible.

12 In 2006 and 2007, a no-claim rebate of €255 was implemented in the Dutch health insurance system but substituted by a mandatory deductible in 2008 since the five largest health insurers and several politicians argued that the no-claim rebate would be unfair to chronically ill and elderly, that it hardly resulted in any restrain on healthcare expenses and that it resulted in a lot of administrative hassle [11]. Research by Holland et al. [21] indicates that the no-claim rebate provided only a weak incentive to reduce healthcare consumption. Furthermore, although seemingly contradictory to the previous statement, their study shows a potential danger of strategic postponement of healthcare utilization (i.e., in order to receive the no-claim rebate), which may have adverse health effects.

13 In the Dutch health insurance market, the average offered premium rebate was €240 for a voluntary deductible of €500 in 2014. If the insured would incur healthcare expenses larger than the (mandatory and) voluntary deductible, the loss would be equivalent to €260. In case of a no-claim rebate, this potential loss is added to the premium (i.e., the premium is increased with €260 compared to the current design of the voluntary deductible). In return for this premium increase, the insured will receive up to €500 (i.e., the original voluntary deductible amount) in return if no or little healthcare expenses are incurred.

14 US dollars are converted to euros using an exchange rate of US$1 = €0.8262 (January 1, 2015).

## References

[1] Attema AE, Brouwer WBF, l’Haridon O (2013). Prospect theory in the health domain: a quantitative assessment. J. Health Econ..

[2] Baicker K, Congdon WJ, Mullainathan S (2012). Health insurance coverage and take-up: lessons from behavioral economics. Milbank Q..

[3] Bateman I, Munro A, Rhodes B, Starmer C, Sugden R (1997). A test of the theory of reference-dependent preferences. Q. J. Econ..

[4] Bateman I, Kahneman D, Munro A, Starmer C, Sugden R (2005). Testing competing models of loss aversion: an adversarial collaboration. J. Public Econ..

[5] Bleichrodt H, Pinto JL, Wakker PP (2001). Making descriptive use of prospect theory to improve the prescriptive use of expected utility. Manag. Sci..

[6] Brook RH, Ware JE, Rogers WH, Keeler EB, Davies AR, Donald CA, Goldberg GA, Lohr KN, Mastay PC, Newhouse JP (1983). Does free care improve adults’ health? Results from a randomized controlled trial. New. Eng. J. Med..

[7] Camerer C, Weber M (1992). Recent developments in modeling preferences: uncertainty and ambiguity. J. Risk Uncertain..

[8] Chetty R, Szeidl A (2007). Consumption commitments and risk preferences. Q. J. Econ..

[9] Chow CC, Sarin RK (2001). Comparative ignorance and the Ellsberg paradox. J. Risk Uncertain..

[10] Ellsberg D (1961). Risk, ambiguity, and the savage axioms. Q. J. Econ..

[11] Elsevier.: Zorgverzekeraars voor afschaffing no-claim. http://www.elsevier.nl/Nederland/nieuws/2006/3/Zorgverzekeraars-voor-afschaffing-no-claim-ELSEVIER072337W/ (2006). (in Dutch)

[12] Friedman M, Savage J (1948). The utility analysis of choice involving risk. J. Polit. Econ. LVI.

[13] Frisch D, Baron J (1988). Ambiguity and rationality. J. Behav. Decis. Mak..

[14] Folland S, Goodman AC, Stano M (2010). The economics of health and health care.

[15] Fox CR, Weber M (2002). Ambiguity aversion, comparative ignorance, and decision context. Organ. Behav. Hum. Decis. Process..

[16] Gorter, J., Schilp, P.: DNB working paper No. 338. Risk preferences over small stakes: evidence from deductible choice. De Nederlandse Bank NV, Amsterdam (2012)

[17] Heath C, Tversky A (1991). Preference and belief: ambiguity and competence in choice under uncertainty. J. Risk Uncertain..

[18] Heath C, Soll JB (1996). Mental budgeting and consumer decisions. J. Consum. Res..

[19] Hershey JC, Kunreuther HC, Schoemaker PJH (1982). Sources of bias in assessment procedures for utility functions. Manag. Sci..

[20] Hershey JC, Schoemaker PJH (1985). Probability versus certainty equivalence methods in utility measurement: are they equivalent?. Manag. Sci..

[21] Holland J, van Exel NJA, Schut FT, Brouwer WBF (2009). Some pain, no gain: experiences with the no-claim rebate in the Dutch health care system. Health Econ. Policy Law.

[22] Hsu J, Reed M, Brand R, Fireman B, Newhouse JP, Selby JV (2004). Cost-sharing: patient knowledge and effects on seeking emergency department care. Med. Care.

[23] Hurley JE, Guindon GE, Jonsson E, Lu M (2008). Medical savings accounts: promises and pitfalls. Financing health care: new ideas for a changing society.

[24] Hurley J (2013). User charges for health care services: some further thoughts. Health. Econ. Policy Law.

[25] Johnson EJ, Hershey J, Meszaros J, Kunreuther H (1993). Framing, probability distortions, and insurance decisions. J. Risk Uncertain..

[26] Kahneman D, Tversky A (1979). Prospect theory: an analysis of decision under risk. Econometrica.

[27] Kahneman D, Miller DT (1986). Norm theory: comparing reality to its alternatives. Psychol. Rev..

[28] Katona G (1975). Psychological economics.

[29] Keppe H-J, Weber M (1995). Judged knowledge and ambiguity aversion. Theory Decis..

[30] Kling JR, Mullainathan S, Shafir E, Vermeulen LC, Wrobel MV (2012). Comparison friction: experimental evidence from Medicare drug plans. Q. J. Econ..

[31] Krieger M, Felder S (2013). Can decision biases improve insurance outcomes? An experiment on status quo bias in health insurance choice. Int. J. Environ. Res. Public Health.

[32] Liebman, J.B., Zeckhauser, R.J.: Schmeduling. Harvard University Working Paper (2004)

[33] Low L, Yee Y, Low D (2012). Using behavioural insights to improve individual health decisions. Behavioural economics and policy design: examples from Singapore.

[34] Markowitz H (1952). The utility of wealth. J. Polit. Econ..

[35] Marquis MS (1981). Consumers’ knowledge about their health insurance coverage.

[36] Moshinsky A, Bar-Hillel M (2010). Loss aversion and status quo label bias. Soc. Cogn..

[37] Newhouse JP, Rand Corporation. Insurance Experiment Group (1993). Free for all?: lessons from the RAND Health Insurance Experiment.

[38] Novemsky N, Kahneman D (2005). The boundaries of loss aversion. J. Mark. Res..

[39] Novemsky N, Kahneman D (2005). How do intentions affect loss aversion?. J. Mark. Res..

[40] Nyhus EK, Webley P, Altman M (2006). Discounting, self-control, and saving. Handbook of contemporary behavioural economics: foundations and developments.

[41] Pauly MV (1968). The economics of moral hazard: comment. Am. Econ. Rev..

[42] Pratt JW (1964). Risk aversion in the small and in the large. Econometrica.

[43] Prelec D, Loewenstein G (1998). The red and the black: mental accounting of savings and debt. Mark. Sci..

[44] Reed M, Fung V, Price M, Brand R, Benedetti N, Derose SF, Newhouse JP, Hsu J (2009). High-deductible health insurance plans: efforts to sharpen a blunt instrument. Health Aff..

[45] Reitsma-van Rooijen, M., Brabers, A.E.M., de Jong, J.D.: Veel zorggebruikers verwachten belemmeringen voor noodzakelijk zorggebruik bij een verplicht eigen risico van 350 euro. NIVEL, Utrecht (2012) (in Dutch)

[46] Rice T (2013). The behavioural economics of health and health care. Annu. Rev. Public Health.

[47] Ritov I, Baron J (1990). Reluctance to vaccinate: omission bias and ambiguity. J. Behav. Dec. Mak..

[48] Ritov I, Baron J (1992). Status-quo and omission biases. J. Risk Uncertain..

[49] Rothschild M, Stiglitz JE (1976). Equilibrium in competitive markets: an essay on the economics of imperfect information. Q. J. Econ..

[50] Samuelson W, Zeckhauser R (1988). Status-quo bias in decision making. J. Risk Uncertain..

[51] Schut FT, van de Ven WPMM (2011). Effects of purchaser competition in the Dutch health system: is the glass half full or half empty?. Health Econ. Policy Law.

[52] Schmidt, U.S.: Insurance demand under prospect theory: a graphical analysis. Kiel Working Paper, No. 1764 (2012)

[53] Schoemaker PJH, Kunreuther HC (1979). An experimental study of insurance decisions. J. Risk Insur..

[54] Smith PC (2013). Universal health coverage and user charges. Health Econ. Policy Law.

[55] Strombom BA, Buchmueller TC, Feldstein PJ (2002). Switching costs, price sensitivity and health plan choice. J. Health Econ..

[56] Sydnor J (2010). (Over)insuring modest risks. Am. Econ. J. App. Econ..

[57] Thaler R (1980). Toward a positive theory of consumer choice. J. Econ. Behav. Organ..

[58] Thaler R (1985). Mental accounting and consumer choice. Mark. Sci..

[59] Thaler RH (1999). Mental accounting matters. J. Behav. Decis. Mak..

[60] Thaler RH, Benartzi S (2004). Save More Tomorrow: using behavioral economics to increase employee saving. J. Polit. Econ..

[61] Thaler RH, Sunstein CR (2009). Nudge: improving decisions about health, wealth and happiness.

[62] Tversky A, Kahneman D (1992). Advances in prospect theory: cumulative representation of uncertainty. J. Risk Uncertain..

[63] Tversky A, Fox CR (1995). Weighing risk and uncertainty. Psychol. Rev..

[64] Van Kleef RC, van de Ven WPMM, van Vliet RCJA (2006). A voluntary deductible in social health insurance with risk equalization: “community-rated or risk-rated premium rebate?”. J. Risk Insur..

[65] Van Winssen KPM, van Kleef RC, van de Ven WPMM (2015). How profitable is a voluntary deductible in health insurance for the consumer?’. Health Policy.

[66] Vektis: Verzekerdenmobiliteit en keuzegedrag: dé feiten over de marktontwikkelingen na invoering van de basisverzekering. Vektis, Zeist (2006) (in Dutch)

[67] Vektis: Zorgthermometer: verzekerden in beweging 2014. Vektis, Zeist (2014) (in Dutch)

[68] Von Neumann J, Morgenstern O (1944). Theory of Games and Economic Behaviour.

[69] Wakker PP, Timmermans DRM, Machielse I (2007). The effects of statistical information on risk and ambiguity attitudes, and on rational insurance decisions. Manag. Sci..

[70] Wakker PP (2010). Prospect theory: for risk and ambiguity.

[71] Wicker FW, Hamman D, Hagen AS, Reed JL, Wiehe JA (1995). Studies of loss aversion and perceived necessity. J. Psychol..

[72] Wilkinson N (2008). An introduction to behavioural economics.

[73] Zweifel P, Manning W, Culyer AJ, Newhouse JP (2000). Moral hazard and consumer incentives in health care. Handbook of Health Economics.

[74] Zweifel P, Breuer M (2006). The case for risk-based premiums in public health insurance. Health Econ. Policy Law.

